# Clinical manifestations and laboratory findings in patients coinfected with dengue virus and SARS-CoV-2: a systematic review

**DOI:** 10.1186/s12879-026-13752-2

**Published:** 2026-06-12

**Authors:** Rahadhyan Adnyanaschah, Rizky Abdulah, Monika Pury Oktora

**Affiliations:** 1 Faculty of Pharmacy, Universitas Padjadjaran, Sumedang, Indonesia; 2 Department of Pharmacology and Clinical Pharmacy, Faculty of Pharmacy, Universitas Padjadjaran, Sumedang, Indonesia; 3 Department of Pharmacology and Clinical Pharmacy, Universitas Padjadjaran, Jl. Raya Bandung Sumedang KM 21, Jatinangor, Sumedang, 45363 Indonesia

**Keywords:** Dengue, COVID-19, Coinfection, Immunological homeostasis

## Abstract

**Background:**

Dengue Hemorrhagic Fever and Coronavirus disease 2019 (COVID-19) are infectious diseases caused by dengue virus (DENV) and severe acute respiratory syndrome coronavirus 2 (SARS-CoV-2). While DENV remains endemic in many tropical regions, SARS-CoV-2 spread globally beginning in 2020. Reports of coinfection have emerged from several countries. This systematic review describes the clinical manifestations and laboratory findings reported in patients coinfected with DENV and SARS-CoV-2.

**Methods:**

This review followed the PRISMA guidelines and collecting data from PubMed for articles published between 2020 and 2025 that discussed DENV and SARS-CoV-2 coinfection. Eligible study designs included theoretical articles, case reports, case series, and cohort studies. Data were synthesized descriptively due to heterogeneity and the absence of comparator groups.

**Results:**

Fifteen studies met the inclusion criteria, identifying 65 patients with SARS-CoV-2 and DENV coinfection. Reported symptoms included fever (69%), vomiting (57%), abdominal pain (55%), rash (54%), and shock (51%). Laboratory findings also showed hematological and physiological alterations including hypotension (57.14%), tachypnea (50%), fever (62.5%), neutropenia (33.33%), neutrophilia (50%), lymphopenia (42.86%), and lymphocytosis (28.57%). These percentages represent descriptive counts across heterogeneous case reports and do not reflect prevalence estimates.

**Conclusions:**

Patients with DENV and SARS-CoV-2 coinfection exhibit a range of clinical and laboratory abnormalities, but the available evidence—primarily case reports and case series without comparator groups—does not allow conclusions about disease severity or prognosis relative to monoinfection. The findings should therefore be interpreted as descriptive and hypothesis-generating. Careful diagnostic evaluation and clinical monitoring remain essential in suspected coinfection cases.

**Clinical trial number:**

Not applicable.

**Supplementary Information:**

The online version contains supplementary material available at 10.1186/s12879-026-13752-2.

## Background

Dengue hemorrhagic fever (DHF) is an infectious disease caused by the dengue virus (DENV), which is transmitted to humans through mosquitoes. This virus is widely distributed and has become endemic in several countries, particularly in tropical regions. Southeast Asia, as a tropical region, reports over 21.000 DHF cases annually in each country [1]. The primary vectors for dengue transmission are *Aedes* species., especially a female *Aedes aegypti* and *Aedes albopictus*. Once infected, individuals typically experience fever lasting about seven days, accompanied by vomiting, rash, myalgias, arthralgias, or other symptoms [2, 3].

While DENV infection continued to persist as an endemic disease, a new kind of respiratory infection disease emerged in 2019 severe acute respiratory syndrome coronavirus 2 (SARS-CoV-2). Unlike DENV, SARS-CoV-2 infection does not require a vector for transmission. Instead, it spreads via airborne particles, enabling more rapid dissemination. The symptoms of SARS-CoV-2 infections differ from DENV, such as sore throat, loss of smell (anosmia), and altered taste (dysgeusia). By November 2020, this virus has spread from China to over 215 countries and territories worldwide [4]. The people infected by this virus were diagnosed with COVID-19 (Coronavirus disease 2019) [5, 6].

Despite the different pathopysiological mechanisms of DHF and COVID-19, coinfection is possible. In such cases, overlapping symptoms—such as fever, headache, and nausea—may occur. Although these viruses affect the body through different pathways, coinfection may exacerbate disease severity. Common symptoms observed in both DHF and COVID-19 include fever, myalgia, arthralgia, headache, lethargy, abdominal pain, diarrhea, vomiting, and sore throat [7]. Current data suggest a fatality rate of approximately 10% for SARS-CoV-2 infection and 8.7% for DENV infection. However, coinfection with both viruses has been associated with a higher fatality rate of 19.1% [8–12].

The overlapping symptoms of DENV and SARS-CoV-2 infections can complicate diagnosis and treatment, leading to delays and potential mismanagement of conditions. Therefore, understanding the interplay between two infections is important to address the consequences on patient’s clinical condition and overall health. This systematic review aims to identify the potential problems faced by patients coinfected with DENV and SARS-CoV-2.

## Methods

We conducted a systematic review by screening out published articles in the PubMed database from 2020 to 2025 using a combination of keywords “dengue hemorrhagic fever”, “post COVID-19”, “after COVID-19”, “sign”, “symptoms”, and “cross reaction” (Appendix 1). Preferred Reporting Items for Systematic Reviews and Meta Analyses (PRISMA) Guideline and Checklist was used for the search process and to guide reporting [13].

Articles addressing DENV and SARS-CoV-2 coinfection were included based on the predefined inclusion criteria. Theoretical studies, case reports, case series, and cohort studies are eligible for inclusion. Theoretical studies provided insights into what might theoretically occur during coinfection, while case reports, case series, and cohort studies are presented the actual possible condition in patients with coinfection.

Reviews, comments, study protocols, opinions, and editorials were excluded. In the process of title and abstract screening, articles not written in English were excluded. Studies not addressing DENV and SARS-CoV-2 coinfection, as well as those reporting negative test results for either infection, were excluded to ensure the inclusion of relevant and accurate data.

Two authors (R.A and R.Ab) developed the search strategy and independently screened titles and abstracts. Potentially eligible articles were reviewed as full texts and assessed for final inclusion by R.A., with R.Ab verified the final inclusion. Additionally, the snowballing method was used to review the reference lists of included articles in order to find relevant additional articles.

Data extraction included study design, study period, country or region, patient age, sample size, and reported symptoms of DHF and COVID-19. Clinical parameters such as heart rate, blood pressure, respiratory rate, oxygen saturation, and body temperature were also recorded. Furthermore, we extracted results from laboratory findings on DENV and SARS-CoV-2 coinfection (i.e. haemoglobin level, white blood cell count, red blood cell count, platelet count, and other relevant markers).

Data synthesis of the laboratory findings and organ function changes was conducted descriptively to summarize the clinical and laboratory features reported in patients with DENV and SARS-CoV-2 coinfection. Abnormal results (either higher or lower than normal) were analyzed to explore their potential clinical relevance and association with reported clinical manifestations in coinfected patients. However, no standardized severity scoring, comparator groups, weighting, meta-analysis, or statistical modeling was performed due to the heterogeneity and descriptive nature of the included studies, which primarily consisted of case reports and case series.

## Results

**Fig. 1:**
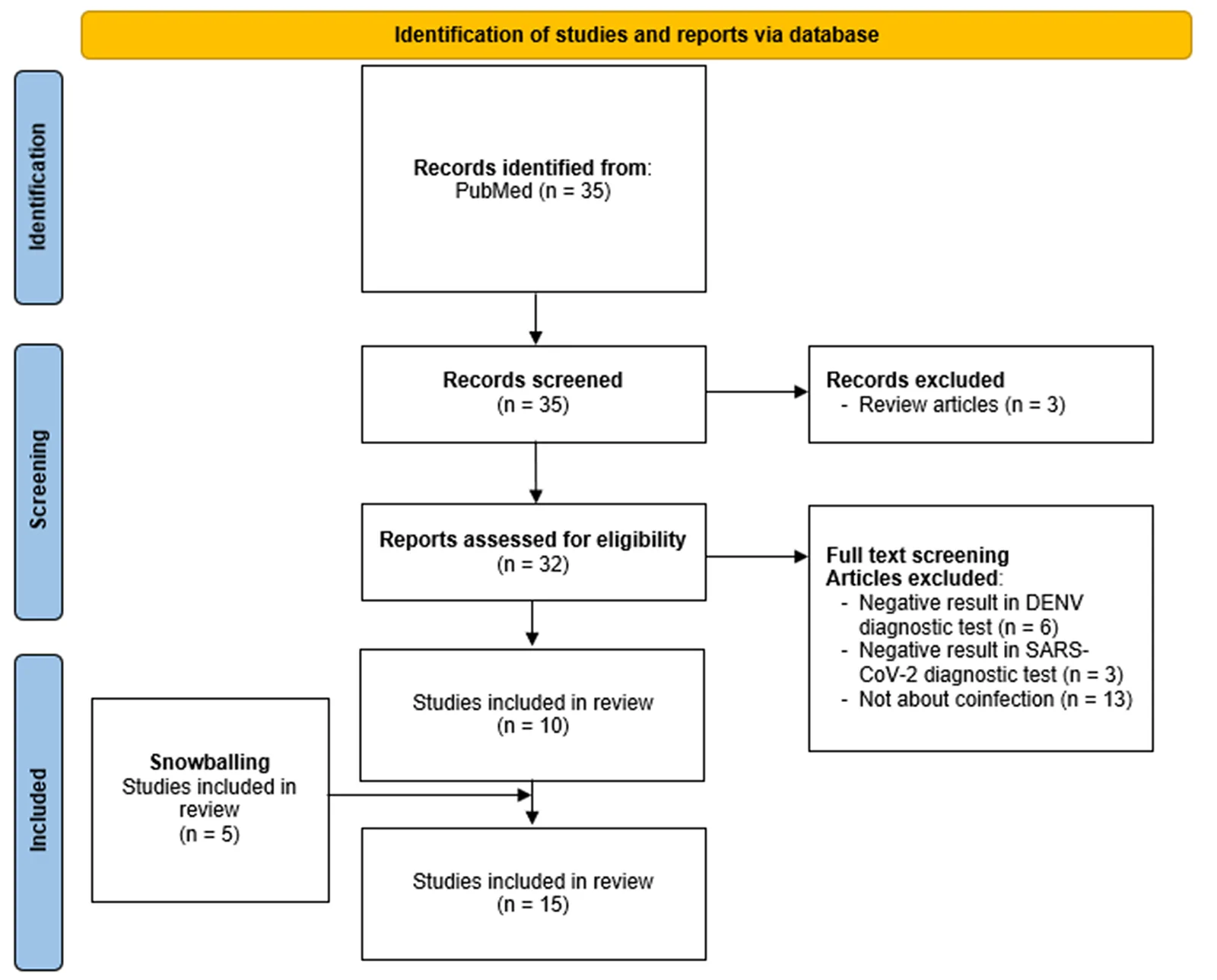
Flow diagram of articles screening

Of the 35 screened articles, ten met the inclusion criteria. In addition, five relevant articles were identified through reference screening of excluded studies. In total, 15 articles were included in the review (Fig. 1).

**Fig. 2:**
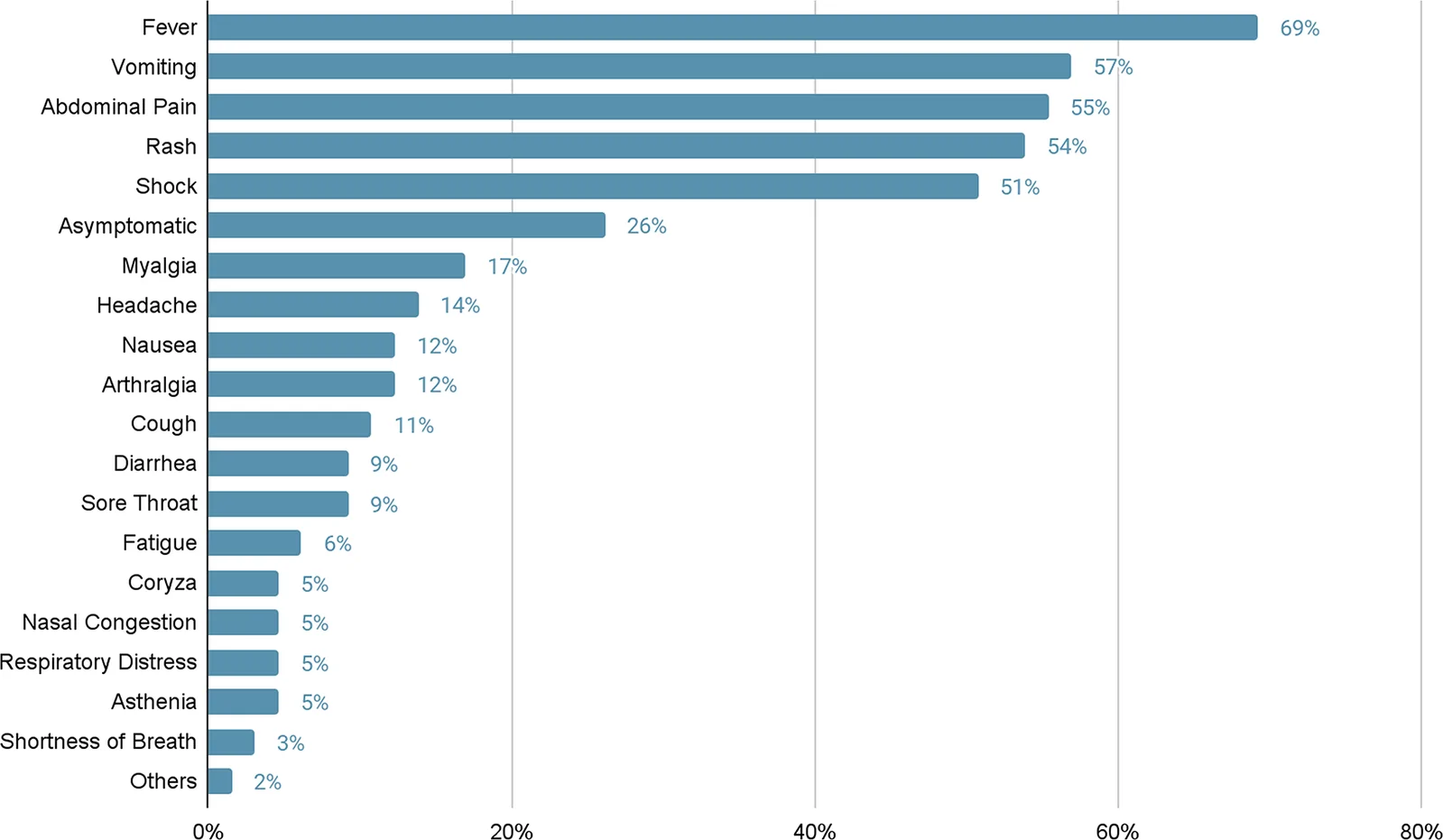
Percentage of symptoms shows on coinfection patient

The included studies comprised one theoretical article, one cohort study, three case series articles, and ten case reports. The studies originated from the Asian region (Indonesia, Thailand, India, and Maldives) and the American region (the United States of America, Mexico, and Brazil). Patient ages range from five months to 75 years, with a total 65 patients included. A wide range of symptoms was reported among patients with DENV and SARS-CoV-2 coinfection. Study characteristics and reported symptoms are summarized in Table 1. All the percentage calculations are used as descriptive counts across the heterogeneous case reports patients criteria. The most frequently reported symptoms were fever (69%), vomiting (57%), abdominal pain (55%), rash (54%), and shock (51%) (Fig. 2). The theoretical article mentioned in the table are excluded from the percentage calculation as it only showed as information and without any patient’s data.

**Table 1:** Study characteristics and symptoms of DENV and SARS-CoV-2 coinfection

Reference	Type of Study	Study Period	Nation or Region	Number of Patients	Age	Symptoms
Machado & Kimura, 2022 [40]	Case series	September 2020 - August 2021	Brazil	3	27–75 years	**General**: fever, myalgia, arthralgia, headache, nausea, vomiting, diarrhea, abdominal pain, asthenia **Respiratory**: cough, sore throat, coryza, nasal congestion, respiratory distress
Sookaromdee & Wiwanitkit, 2021 [41]	Theoretical article	n.a	Indochina	n.a	n.a	Mimicry skin lesion, petechiae, skin rash
Nair, 2021 [42]	Case report	n.a	Southeast Asia	1	62 years	Fever, chills, rigors, arthralgia, myalgia, pinpoint rash over the chest and abdomen
Balasubramani et al., 2022 [43]	Cohort study	October - November 2021	India	31	< 14 years	**Severe**: fever, vomit, abdominal pain, skin rash, shock on arrival **Non-severe**: fever, vomit, abdominal pain
Kariyappa et al., 2021 [44]	Case report	August 2020 - February 2021	India	1	5 months	Shock, fever, pulmonary edema, hepatomegaly
Puri et al., 2022 [45]	Case report	n.a	USA	1	63 years	Fatigue, cough, mild shortness of breath, fever, skin rash
Santa-Cruz et al., 2022 [46]	Case series	April 2020 - March 2021	Brazil	17	18–65 years	None (asymptomatic)
Desdiani, 2022 [47]	Case report	n.a	Indonesia	1	34 years	Shortness of breath, cough, fever, sore throat, myalgia, fatigue, headache, rhinorrhea, diarrhea
Krueger et al., 2021 [48]	Case series	n.a	Brazil	1	16 years	Diarrhea, paresthesia, progressive difficulty to walk, acute and progressive paraparesis
Nasomsong et al., 2021 [29]	Case report	March - April 2020	Thailand	1	50 years	Acute fever, myalgia, nausea, vomiting
Reyes-Ruiz et al., 2021 [49]	Case report	August 2021	Mexico	1	42 years	Fever, headache, diarrhea, chest pain, chills, odynophagia, myalgia, arthralgia, general malaise, itching, low back pain, nausea, loss of appetite, sweating, hyperemia, petechial rash, exanthema
Masyeni et al., 2021 [50]	Case report	n.a	Indonesia	3	24, 59, and 69 years	**Case 1**: myalgia, headache arthralgia, retro-orbital pain, nausea, vomiting, **Case 2**: continuous fever, dry cough, sore throat, myalgia, headache, arthralgia, nausea **Case 3**: myalgia, arthralgia, nausea, mild cough
Hilmy et al., 2021 [20]	Case report	n.a	Maldives	2	39 and 38 years	**Case 1**: persistent high-grade fever, retro-orbital headache, fatigue, myalgia, right upper quadrant pain, vomiting, loose stools **Case 2**: intermittent fever, generalized headache, sore throat, dysgeusia, anosmia
Somasetia et al., 2020 [51]	Case report	April 2020	Indonesia	1	6 years	Fatigue, looks pale, shock, fever, abdominal pain
Yuliarto, 2021 [19]	Case report	n.a	Indonesia	1	7 years	High fever, abdominal pain, non-pitting edema, erythematous rash

Among the included studies, nine reported data on organ function changes in coinfected patients (Table 2). Heart rate ranged from 80 to 135 bpm; systolic blood pressure from 70 to 145 mmHg and diastolic from 40 to 90 mmHg; respiratory rate from 16 to 40 breaths per minute; oxygen saturation from 83% to 99%; and body temperature from 36.5 °C to 39 °C. Based on the available data, patients with coinfection had lower values for blood pressure (57.14%), higher respiratory rate (50%) and higher body temperature (62.50%) than normal (Appendix 2).

Ten studies reported laboratory findings of DENV and SARS-CoV-2 coinfection (Table 3). Hemoglobin level ranged from 2.9 to 18.3 g/dL, hematocrit from 9.3 to 54%, white blood cells count from 1.02 × 10^3^/µL to 18 × 10^3^/µL, red blood cells count from 1.13 × 10^6^/µL to 5.17 × 10^6^/µL, and platelet count from 17 × 10^3^/µL to 370 × 10^3^/µL. Differential counts included neutrophils (14.7–92.1%), lymphocytes (3–67.1%), monocytes (3–20%), eosinophils (0–1%), and basophils (0–1%). Overall, coinfected patients commonly showed decreased hemoglobin (42.86%), increased white blood cell counts (64.52%), decreased red blood cell counts (50%), increased neutrophil counts (50%), and decreased lymphocyte counts (42.86%) (Appendix 2).

## Discussion

Patients coinfected with DENV and SARS-CoV-2 commonly experienced symptoms such as fever, vomiting, abdominal pain, rash, and shock. These patients also showed lower blood pressure, higher respiratory rate, elevated body temperature, and abnormal blood counts, including changes in lymphocytes, neutrophils, and hemoglobin level. However, because the included studies did not include comparator groups with dengue or COVID-19 monoinfection, these findings should be interpreted as descriptive observations rather than evidence of increased severity caused by coinfection.

Given the known serological cross-reactivity between DENV and SARS-CoV-2, diagnostic uncertainty may influence case attribution and symptom interpretation. False-positive dengue serology in COVID-19 patients—and vice versa—has been documented, meaning that some reported coinfections may reflect diagnostic overlap rather than true concurrent infection.

Fever and myalgia were among the most frequently reported symptoms in coinfected patients. Notably, no evidence of pulmonary infection was observed, and chest imaging results were typically normal. The absence of abnormalities in chest imaging highlights the importance of relying on laboratory results and monitoring hospitalization time for clearer and safer patient management [14]. To our knowledge, no prior information has been available on organ function changes or laboratory findings associated with this coinfection.

Abnormal counts of lymphocytes, neutrophils, and hemoglobin may reflect disturbances in immune regulation and inflammatory responses during coinfection. When the homeostasis stage is disturbed, it may lead to conditions such as chronic inflammation, autoimmune diseases, immunodeficiency, frequent infections, or even cancers [15]. However, these mechanisms were not directly measured in the included studies and therefore remain theoretical interpretations. Accordingly, this review cannot determine whether these abnormalities are more severe than those observed in dengue or COVID-19 monoinfection.

### Hypotension

Previous findings showed that hypotension might occur in DHF patients but it typically seen in patients under 12 years old [16]. In DHF patients, hypotension often coincides with the elevated lymphocyte levels, which may indicate an active immune response [16]. Hypotension has also been reported in COVID-19 patients, although it is relatively rare [17]. Low blood pressure can increase the risk of mortality in these patients [17]. It reduces blood flow to immune organs impairing the body’s ability to fight infections [18]. The correlation of clinical manifestation on cases collected showed that hypotension and lymphocytosis occur together in the same patients [19, 20].

### Tachypnea

Tachypnea is a condition characterized by an abnormally high respiratory rate. Normally, human respiratory rate should be around 12 to 20 breaths per minute [21]. However, COVID-19 patients may develop tachypnea with a respiratory rate exceeding 20 breaths per minute [22]. Similarly, DHF patients can also cause tachypnea with respiratory rate in the same range. In DHF, tachypnea commonly appears in patients older than 12 years, while those under 12 are less likely to experience it [16]. These observations suggest that patients coinfected with DENV and SARS-CoV-2 may have a higher risk of developing tachypnea compared to those with a single infection.

### Fever

Fever is the body’s systemic response to infections, with elevated temperature serving as a defense mechanism to enhance survivability and fight infections [23]. In DHF patients, fever usually lasts around 24 h during the first day of febrile phase, followed by another temperature elevation that lasts for at least a day after [3]. Fever is also common in COVID-19, though it has been linked to a potential worsening infection prognosis [24]. However, further research is needed to confirm if fever is a reliable marker of severe injury in COVID-19 [24]. Correlating these factors, the presence of fever in coinfected patients may compromise the body’s defence mechanism, increasing the risk of severe complication.

### Neutropenia

The normal neutrophil count in blood ranges from 40% to 73%. Neutropenia occurs when neutrophil levels drop below 40% [25]. Viral infections, such as, herpes viruses, hepatitis viruses, rubella, measles virus, chicken pox virus, and respiratory viruses like RSV, influenza A, influenza B, and parainfluenza can lead to neutropenia. Previous study revealed that SARS-CoV-2 and DENV can also induce neutropenia by impairing the growth and differentiation of bone marrow [26].

Neutropenia is considered a life-threatening complication in both COVID-19 and DHF patients, as it can lead to condition like leukocyte adhesion deficiency syndromes, chronic granulomatous disease, Shwachman-Diamond syndrome, and Chediak-Higashi syndrome, potentially worsening the patient’s condition [27, 28]. In three reported cases, neutropenia occurred simultaneously with fever [20, 29].

### Neutrophilia

Neutrophilia is the opposite of neutropenia and occurs when neutrophil counts exceed 73% [25]. This condition, also known as secondary neutrophilia can be triggered by viral infection [30]. An elevated neutrophil count in patients with virus infection suggests greater clinical burden and worse prognosis [31]. In patients with SARS-CoV-2, higher neutrophil levels indicate more severe [31]. Similarly, in DHF patients, elevated neutrophil counts are linked to a higher of DENV transmission [32]. Thus, in patients coinfected with SARS-CoV-2 and DENV, neutrophilia indicates a poorer prognosis and higher transmission possibilities.

### Lymphopenia

Lymphopenia or lymphocytopenia occurs when lymphopenia, or lymphocytopenia, occurs when lymphocyte levels drop below the normal range of 19–48%. Levels under 19% can impair immune responses and lead to systemic immunosuppression, which is strongly associated with adverse outcomes and higher risk of fatal events [25, 33].

There are many types of viruses that can reduce lymphocyte levels in the host’s blood, such as Coronaviridae, Retroviridae, Filoviridae, Orthomyxoviridae, Paramyxoviridae, Pneumoviridae, Arteriviridae, Picornaviridae, Flaviviridae, Caliciviridae, Arenaviridae, Togaviridae, Asfarviridae, Circoviridae, Herpesviridae, Alphaherpesvirinae, and Parvoviridae. Both SARS-CoV-2 or DENV are also included in the viruses that cause lymphopenia. While monoinfection can cause lymphopenia, coinfection with both viruses may exacerbate the condition due to further weakening of the body’s defense system [34]. It has also been previously noted that lymphopenia is associated with a poor prognosis in infected patients [35].

### Lymphocytosis

Lymphocytosis is characterized by an elevated lymphocyte count, typically exceeding 48% [25]. Viral infections such as Epstein-Barr Virus, Cytomegalovirus, HIV, influenza, hepatitis, mumps, measles, rubella, human T lymphocytic virus type 1 (HTLV-1), and adenovirus, are known to cause lymphocytosis in patients [36]. The SARS-CoV-2 virus are not included as one of lymphocytosis causes, but since the cases are showing a coinfection with DENV, the possibility of lymphocytosis event is elevated that might cause worse conditions on patients.

Elevated lymphocyte levels trigger activation of immune cells and overproduction of proinflammatory cytokines, which can cause myalgia [37]. Myalgia, a form of muscle aches, can impair patient’s daily functioning and can cause muscle cramp with varying recovery stages [38]. Clinical cases have shown that lymphocytosis often coincides with myalgia [20, 29].

### Strength and limitations

This systematic review summarizes cases of SARS-CoV-2 and DENV coinfection in patients with varying conditions. By pooling results from multiple studies, we aimed to provide a broader understanding of the relationship between SARS-CoV-2 and DENV coinfection. Although this area remains relatively understudied, examining these infections together offers valuable insights into how they may interact and potentially exacerbate clinical outcomes.

A key limitation of this review is that not all included studies provided complete data. Eight articles lacked essential information, so the assessment of symptom severity was primarily based on presenting symptoms. Data on laboratory findings and changes in organ function were used to support these assessments and help draw conclusion of the potential worsening symptoms in coinfected patients. However, symptoms in both SARS-CoV-2 and DHF can vary, and inconsistent reporting may lead to some variability in the findings. Furthermore, due to the absence of comparator groups involving patients with dengue or COVID-19 monoinfection, the included studies did not allow for direct evaluation of differences in disease severity, prognosis, or clinical risk associated with coinfection. As a result, no direct comparative assessment of disease severity, prognosis, or clinical risk associated with coinfection could be performed. Therefore, the findings should be interpreted as descriptive summaries of reported clinical and laboratory features rather than evidence that coinfection results in worse outcomes compared with either infection alone.

In addition, the overall number of eligible studies was small, and most of the included literature consisted of case reports and case series. This limits the generalizability of the findings, as case reports often lack standardized reporting, comprehensive clinical data, and consistent follow-up. The predominance of such study designs reduces the strength of the evidence and restricts the ability to draw broader or more definitive conclusions about coinfection patterns. although most included studies were case reports, they offer detailed, patient-level information that helps identify patterns that may not yet appear in larger epidemiological studies. Furthermore, we did not perform weighting, meta-analysis, or any form of statistical inference. This was intentional, as the available studies consist primarily of isolated case reports and small case series, lack standardized reporting, and do not provide comparable denominators or control groups.

This systematic review was not registered in PROSPERO, as the protocol was still being refined and data extraction had already begun. Nevertheless, established systematic review methods were followed, including a predefined search strategy, clear inclusion and exclusion criteria, and transparent reporting to ensure rigor and reproducibility. Finally, because the search was limited to PubMed, studies indexed in other databases such as Scopus, Web of Science, or Embase may not have been captured, which represents a potential source of selection bias.

### Future perspective

This study identifies patterns, common symptoms, and manifestations in coinfection cases, which can help improve management and prevention strategies. Prompt health interventions are crucial for patients with SARS-CoV-2 and DENV coinfection, especially for symptoms like hypotension, tachypnea, neutropenia, neutrophilia, lymphopenia, and lymphocytosis. Appropriate health management and intervention for coinfection patients are crucial to reducing mortality rate. While medications like Favipiravir, Hydroxychloroquine, and Azitromycin were historically reported on early-pandemic experimental use, they are insufficient to prevent further transmission and mitigate the other severe complications mentioned earlier [12].

Future research may discover more about the potential absence of symptoms, which could represent another form of abnormality caused by coinfection. Identifying these hidden symptoms is important to prevent delayed intervention. Currently, raising public awareness is the primary preventive measure against coinfection worldwide [39]. However, awareness alone is not enough to prevent its spread. The findings of this review can provide valuable insight for healthcare policymakers and practitioners to enhance triage, treat, and prevent severe outcomes in coinfected patients.

## Conclusion

Patients coinfected with SARS-CoV-2 and DENV exhibited a range of clinical manifestations and laboratory abnormalities, including fever, vomiting, abdominal pain, shock, hypotension, tachypnea, altered hemoglobin levels, and abnormal neutrophil and lymphocyte counts. These findings may reflect complex inflammatory and immune responses during coinfection and may complicate clinical management. However, because the available evidence consisted primarily of case reports and case series without comparator groups, this review cannot determine whether coinfection is associated with greater severity, poorer prognosis, or higher risk compared with dengue or COVID-19 monoinfection. Therefore, the findings should be interpreted as descriptive and hypothesis-generating. Despite some data limitations across studies, this review highlights the importance of careful diagnosis, monitoring, and supportive management in patients with suspected SARS-CoV-2 and DENV coinfection. Future comparative and large-scale studies are needed to better understand the clinical implications and outcomes of coinfection.

**Table 2:** Organ function changes of DENV and SARS-CoV-2 Coinfection

Reference	Heart Rate (bpm)	Blood Pressure (mmHg)	Respiratory Rate (bpm)	Oxygen Saturation (%)	Temperature (^o^C)
Nemcova et al., 2020; Sapra et al., 2024 [21,52]*	60–100	120/70	12–20	96–98	36.5–37.5
Nair, 2021 [42]	91	145/80**	n.a	99**	38.8
Puri et al., 2022 [45]	121**	n.a	n.a	95**	n.a
Desdiani, 2022 [47]	n.a	n.a	n.a	84–92**	n.a
Nasomsong et al., 2021 [29]	82	120/80	16	98	39**
Reyes-Ruiz et al., 2021 [49]	88	130/90**	20	97	37–39**
Masyeni et al., 2021 [50] (Case 3)	n.a	n.a	n.a	n.a	37.8**
Hilmy et al., 2021 [20] Case 1 Case 2	80 84	113/78** 100/60**	20 21**	98 97	37.5 37.4
Somasetia et al., 2020 [51]	135**	70/40**	40**	83**	36.5
Yuliarto, 2021 [19]	120**	94/50**	26**	98	38.9**

*Normal range reference for organ function**Abnormal value

**Table 3:** Laboratory findings of DENV and SARS-CoV-2 coinfection

Reference	Hb (g/dL)	HCT (%)	WBC (10^3^/µL)	RBC (10^6^/µL)	PLT (10^3^/µL)	Other Cells (%)
Seo & Lee, 2022 [25]*	12–17	37–52	4-10.8	4-6.1	150–400	**NT** : 40–73 **LY** : 19–48 **MN** : 0.4–10 **EO** : 0–7 **BS** : 0–2
Machado & Kimura, 2022 [40]	n.a	n.a	> 4	n.a	> 150	n.a
Nair, 2021 [42]	18.3**	54**	4	n.a	111**	n.a
Santa-Cruz et al., 2022 [46]	n.a	n.a	5.6–11.5**	n.a	185–370	n.a
Desdiani, 2022 [47]	n.a	n.a	12.3**	n.a	86**	**NT** : 74.8–92.1** **LY** : 3–12** **MN** : 11–20**
Nasomsong et al., 2021 [29]	10.8**-12.5	33**-39	1.5**-4.4	n.a	162–347	**NT** : 34–89** **LY** : 6–55** **MN** : 3–10 **EO** : 0–1 **BS** : 0–1
Reyes-Ruiz et al., 2021 [49]	16.9–16	42.6–45.2	2**-4	4.83–5.17	120**-230	**LY** : 32.2–48.1 **MN** : 7-8.2
Masyeni et al., 2021 [50] Case 1 Case 2 Case 3	n.a n.a n.a	n.a n.a 43,2	1.97–2.38** 1.02** 3.4**	n.a n.a n.a	108** 82–141** 236	n.a n.a **EO** : 0
Hilmy et al., 2021 [20] Case 1 Case 2	14.8–16.3 14-14.8	39.7–46.9 41.5–45.9	6.9–11.4** 2.8**-8	n.a n.a	193–284 156–236	**NT** : 18.8**-44.3 **LY** : 42.3–65** **NT** : 14.7**-41.9 **LY** : 41.3–67.1**
Somasetia et al., 2020 [51]	2.9**	9.3**	18**	1.13**	213	**NT** : 1 (B), 55 (S) **LY** : 33 **MN** : 4 **EO** : 0 **BS** : 0
Yuliarto, 2021 [19]	9.6**-14.8	26.2–39.1**	5.25–9.54	n.a	17–36**	**NT** : 76.9–86.4** **LY** : 6.8–15.9**

*Normal range reference for laboratory findings**Abnormal value

## Supplementary Information

Below is the link to the electronic supplementary material.

Supplementary Material 1

Supplementary Material 2

## Data Availability

The authors confirm that the data supporting the findings of this study are available within the article and its supplementary materials.

## Abbreviations

B: Band Neutrophil
BS: Basophil
COVID-19: Coronavirus Disease 2019
DENV: Dengue Virus
DHF: Dengue Hemorrhagic Fever
EO: Eosinophil
Hb: Hemoglobin
HCT: Hematocrit
LY: Lymphocyte
MN: Monocyte
NT: Neutrophil
PLT: Platelets Count
RBC: Red Blood Cells
S: Segment Neutrophil
SARS-CoV-2: Severe Acute Respiratory Syndrome Coronavirus 2
WBC: White Blood Cells
