# Supplementary material for: Clinical manifestations and laboratory findings in patients coinfected with dengue virus and SARS-CoV-2: a systematic review
Source: BMC Infect Dis. 2026 Jun 12;26:1527. doi: 10.1186/s12879-026-13752-2 (PMC13487913; doi:10.1186/s12879-026-13752-2)
Supplement: Supplementary file 1 [file 12879_2026_13752_MOESM1_ESM.docx]

**Appendix 1. Search Strategy**

**Databases Searched**

PubMed

**Time restriction:** Last 5 years **Language:** English **Search period:** [Insert date of search]

1. **Pubmed Search Strategy**

("Dengue"[Mesh] OR "Dengue Hemorrhagic Fever"[Mesh] OR dengue OR "dengue hemorrhagic fever" OR DHF)

AND

("COVID-19"[Mesh] OR "SARS-CoV-2"[Mesh] OR COVID-19 OR SARS-CoV-2 OR coronavirus)

AND

(symptom* OR clinical feature* OR "clinical presentation" OR sign* OR "cross reaction" OR "cross-reactivity")

Next database to be searched: Scopus, Web of Science, Embase

2. **Scopus Search Strategy**

(TITLE-ABS-KEY(dengue OR "dengue hemorrhagic fever" OR DHF))

AND

(TITLE-ABS-KEY(COVID-19 OR SARS-CoV-2 OR coronavirus))

AND

(TITLE-ABS-KEY(symptom* OR "clinical presentation" OR sign* OR "cross reaction" OR "cross-reactivity"))

AND

(PUBYEAR 2020-2025

3. **Web of Science**

TS=(dengue OR "dengue hemorrhagic fever" OR DHF)

AND

TS=(COVID-19 OR SARS-CoV-2 OR coronavirus)

AND

TS=(symptom* OR "clinical presentation" OR sign* OR "cross reaction" OR "cross-reactivity")

Refined by: Publication Years = 2020–2025

4. **Embase**

('dengue'/exp OR dengue OR 'dengue hemorrhagic fever' OR DHF)

AND

('covid-19'/exp OR 'sars-cov-2'/exp OR COVID-19 OR SARS-CoV-2 OR coronavirus)

AND

(symptom* OR 'clinical presentation' OR sign* OR 'cross reaction' OR 'cross-reactivity')

AND

[2019-2024]/py
