# Supplementary material for: Clinical manifestations and laboratory findings in patients coinfected with dengue virus and SARS-CoV-2: a systematic review
Source: BMC Infect Dis. 2026 Jun 12;26:1527. doi: 10.1186/s12879-026-13752-2 (PMC13487913; doi:10.1186/s12879-026-13752-2)
Supplement: Supplementary file 2 [file 12879_2026_13752_MOESM2_ESM.docx]

**Appendix 2. Organ function changes and laboratory findings**

**Organ Function Changes**

| Data from 65 patients in included studies | Heart Rate | | Blood Pressure | | Respiratory Rate | | Oxygen Saturation | | Body Temperature | |
| --- | --- | --- | --- | --- | --- | --- | --- | --- | --- | --- |
|  | n | % | n | % | n | % | n | % | n | % |
| Normal | 5 | 7.69 | 1 | 1.54 | 3 | 4.62 | 5 | 7.69 | 3 | 4.62 |
| Abnormal | 3 | 4.62 | 6 | 9.23 | 3 | 4.62 | 4 | 6.15 | 5 | 7.69 |
| Data not available | 57 | 87.69 | 58 | 89.23 | 59 | 90.77 | 56 | 86.15 | 57 | 87.69 |

**Organ Function Changes from Only Available Data**

| From only available data | Heart Rate | | Blood Pressure | | Respiratory Rate | | Oxygen Saturation | | Body Temperature | |
| --- | --- | --- | --- | --- | --- | --- | --- | --- | --- | --- |
|  | n | % | n | % | n | % | n | % | n | % |
| Low | 0 | 0.00 | 4 | 57.14 | 0 | 0.00 | 3 | 33.33 | 0 | 0.00 |
| Normal | 5 | 62.50 | 1 | 14.28 | 3 | 50.00 | 5 | 55.55 | 3 | 37.50 |
| High | 3 | 37.50 | 2 | 28.57 | 3 | 50.00 | 1 | 11.11 | 5 | 62.50 |

**Laboratory Findings**

| Data from 65 patients in included studies | Hemoglobin | | Hematocrit | | White Blood Cell | | Red Blood Cell | | Platelet | | Neutrophil | | Lymphocyte | | Monocyte | | Eosinophil | | Basophil | |
| --- | --- | --- | --- | --- | --- | --- | --- | --- | --- | --- | --- | --- | --- | --- | --- | --- | --- | --- | --- | --- |
|  | n | % | n | % | n | % | n | % | n | % | n | % | n | % | n | % | n | % | n | % |
| Normal | 3 | 4.62 | 4 | 6.15 | 5 | 7.69 | 1 | 1.54 | 25 | 38.46 | 1 | 1.54 | 2 | 3.08 | 2 | 3.08 | 3 | 4.62 | 2 | 3.08 |
| Abnormal | 4 | 6.15 | 4 | 6.15 | 26 | 40.00 | 1 | 1.54 | 6 | 9.23 | 5 | 7.69 | 5 | 7.69 | 2 | 3.08 | 0 | 0.00 | 0 | 0.00 |
| Data not available | 58 | 89.23 | 57 | 87.69 | 34 | 52.31 | 63 | 96.92 | 34 | 52.31 | 59 | 90.77 | 58 | 90.63 | 61 | 93.85 | 62 | 95.38 | 63 | 96.92 |

**Laboratory Findings from Only Available Data**

| Data from 65 patients in included studies | Hemoglobin | | Hematocrit | | White Blood Cell | | Red Blood Cell | | Platelet | | Neutrophil | | Lymphocyte | | Monocyte | | Eosinophil | | Basophil | |
| --- | --- | --- | --- | --- | --- | --- | --- | --- | --- | --- | --- | --- | --- | --- | --- | --- | --- | --- | --- | --- |
|  | n | % | n | % | n | % | n | % | n | % | n | % | n | % | n | % | n | % | n | % |
| Low | 3 | 42.86 | 3 | 37.50 | 6 | 19.35 | 1 | 50.00 | 6 | 19.35 | 2 | 33.33 | 3 | 42.86 | 0 | 0.00 | 0 | 0.00 | 0 | 0.00 |
| Normal | 3 | 42.86 | 4 | 50.00 | 5 | 16.13 | 1 | 50.00 | 25 | 80.65 | 1 | 16.67 | 2 | 28.57 | 3 | 75.00 | 3 | 100.00 | 2 | 100.00 |
| High | 1 | 14.29 | 1 | 12.50 | 20 | 64.52 | 0 | 0.00 | 0 | 0.00 | 3 | 50.00 | 2 | 28.57 | 1 | 25.00 | 0 | 0.00 | 0 | 0.00 |
